# In Vitro Embryos of Romosinuano and Tropical Milking Cattle during Three Seasons in Veracruz, Mexico

**DOI:** 10.3390/ani14131922

**Published:** 2024-06-29

**Authors:** Froylan Rosales-Martínez, Carlos Miguel Becerril-Pérez, Adalberto Rosendo-Ponce, Alberto Riaño-Gaya, César Cortez-Romero, Jaime Gallegos-Sánchez, Salvador Romo-García

**Affiliations:** 1 Facultad Maya de Estudios Agropecuarios, Universidad Autónoma de Chiapas, Catazajá C.P. 29980, Chiapas, Mexico; froylan.rosales@unach.mx; 2 Colegio de Postgraduados, Programa de Agroecosistemas Tropicales, Campus Veracruz, Manlio Fabio Altamirano C.P. 94251, Veracruz, Mexico; arosendo@colpos.mx; 3 Colegio de Postgraduados, Programa de Ganadería, Campus Montecillo, Texcoco C.P. 56230, Estado de México, Mexico; gallegos@colpos.mx; 4 Reproducción Genética Avanzada RGA In Vitro, Boca del Río C.P. 94299, Veracruz, Mexico; rga_in_vitro@hotmail.com; 5 Colegio de Postgraduados, Campus San Luis, Salinas de Hidalgo C.P. 78622, San Luis Potosí, Mexico; ccortez@colpos.mx; 6 Facultad de Estudios Superiores de Cuautitlán, Universidad Nacional Autónoma de México, Cuatitlán Izcalli C.P. 54714, Estado de México, Mexico; sromo_99@yahoo.com

**Keywords:** adapted breeds, climatic change, genetic resources, hot climates, OPU-IVF, tropical region

## Abstract

**Simple Summary:**

Heat stress in bovine females from hot tropical regions is one of the main factors that negatively affects the success of ovum pick-up and in vitro fertilization programs (OPU-IVF). Seasonal climatological variation allows us to differentiate significant physiological effects in donors that affect the quantity and quality of embryos. The results of this study indicated a significant effect of season and breed on cumulus–oocyte complexes (COCs) and embryos, as well as the influence of weight and age, hormonal and physiological conditions of criollo Romosinuano (RM) and Tropical Milking (TM) donors. The greatest quantity and quality of COCs and code one blastocysts was observed in the hot humid and fresh dry seasons, so it is concluded that these seasons are the most suitable to perform OPU-IVF in RM and TM donors.

**Abstract:**

One of the main factors that influences the fertility of cattle in grazing systems in hot tropical climates is heat stress. The objective of this study was to evaluate the effect of season, breed, hormonal and physiological condition on the quantity and quality of cumulus–oocyte complexes (COCs) and embryos produced in vitro, from Romosinuano (RM) and Tropical Milking (TM) donors. Three ovum pick-up and in vitro fertilization (OPU-IVF) were performed, one per season: hot dry (HD; 10, 10), hot humid (HH; 9, 9) and fresh dry (FD; 7, 10) in RM and LT donors. Serum levels of cortisol, insulin and glucose were measured, in addition to heart rate (HR), respiratory rate (RR) and rectal temperature (RT). Effect of season x genotype interaction (*p* ≤ 0.05) was observed in all COC variables and only in cleavage embryos (CLI) (*p* ≤ 0.05). Body weight (BW) affected all COC variables (*p* ≤ 0.01), except unviable (UNV) although affected degenerated embryos (DEG) (*p* ≤ 0.01) and total blastocysts (BLAST) (*p* ≤ 0.01). Cow age only affected viable COCs (VIAB) (*p* ≤ 0.05), code one blastocysts (BC1) and BLAST (*p* ≤ 0.01). Cortisol affected total COCs (COCsT), VIAB and total matured in vitro (TMIV) (*p* ≤ 0.01), as well as CLI, BC1 (*p* ≤ 0.01) and BLAST (*p* ≤ 0.05). Insulin affected COCsT (*p* ≤ 0.01), UNV (*p* ≤ 0.05), denuded oocytes (DE) (*p* ≤ 0.01), BC1 and code two blastocysts (BC2) (*p* ≤ 0.01). Glucose affected all COC variables (*p* ≤ 0.01), except UNV and all embryo variables except BC2. HR affected COCsT, DE, TMIV (*p* ≤ 0.01), CLI, BLAST and DEG (*p* ≤ 0.05). RR affected COCsT, UNV, VIAB, CLI (*p* ≤ 0.05), BC1, BLAST and DEG (*p* ≤ 0.01). RT only affected DE, VIAB (*p* ≤ 0.01) and BLAST (*p* ≤ 0.05). The seasonal climatic year variation of Veracruz and changes in physiological and hormonal variables have diverse effects on the cumulus–oocyte complexes and embryos produced by RM and TM donors.

## 1. Introduction

Ovum pick-up (OPU) and in vitro fertilization (IVF) are used in cattle to accelerate the diaspora of genotypes and genetically superior females [1]; furthermore, they enable the multiplication of small populations adapted to adverse environments and even in danger of extinction [2]. OPU-IVF has been successful for *Bos taurus* breeds in the temperate climatic region and for *Bos indicus* breeds in the hot climatic tropical region [3,4]. However, for *Bos taurus* adapted breeds to the hot climatic tropical region, studies are scarce and less successful [5,6]; among other possible factors, the adverse hot humid climate directly and indirectly affects the reproductive performance of females [7]. The low fertility of cattle in hot climates is related to heat stress caused by high maximum temperatures (Tmax) and relative humidity (RH) [8]. Animals under heat stress increase the concentrations of cortisol, insulin and glucose in blood [9,10]. In addition, they respond physiologically by increasing heart rate, respiratory rate and rectal temperature, among other acclimatization mechanisms, with the aim of dissipating body heat [11,12]; these changes negatively affect reproductive behavior [13].

Although OPU-IVF are reproductive technologies that contribute to rapidly increasing herds of pure animals, inter-annual season variability with Tmax and RH higher than 30 °C and 84% and highly variable rainfall (PP) [14,15] can decrease their success. In recent years, 90% of the annual pluvial precipitation has occurred in the hot humid season; while in the fresh dry and hot dry season, only around 5% has occurred in each, with a total of seven months of drought [16,17,18]. In the female bovine, the increase in body temperature reduces the follicular population and decreases the size of the preovulatory follicles; in addition, a higher degree Celsius in the temperature of the follicles in the ovaries inhibits ovulation [19]. In the oocyte, high temperatures cause damage to cellular organelles and DNA [20]; and in the first stages of embryo development, cellular and epigenetic changes are generated that limit its development and quality [21].

The criollo breeds Romosinuano and Tropical Milking are *Bos taurus* naturalized to hot climates of the tropical region, with populations established in different countries of the Americas, although relatively small with thousands of individuals [22,23,24]. Studies have been carried out to determine the effect of Tmax and RH on the fertility of TM heifers and cows; it was found that the lowest rates of pregnancy, less than 50%, occur in the hot dry season with a Tmax greater than 27 °C and an RH of 85% for heifers [18], but no season effect was observed in cows [25]. When comparing Brahman and Angus breeds to determine the effect of heat shock on oocytes cultured in vitro at 41 °C, oocytes had greater cleavage, with embryos of more than eight cells four days after IVF [26]. The objective of this study was to evaluate the effect of the season of the year, the breed, the hormonal and physiological condition of cows on the quantity and quality of the cumulus–oocyte complexes and embryos produced in vitro of Romosinuano and Tropical Milking females.

## 2. Materials and Methods

### 2.1. Characterization of the Seasons of the Year

The study was carried out in three climatic seasons (EST): hot dry (HD, March–May, with Tmax, RH and PP of 31.6 ± 1.0 °C, 77.2 ± 1.1% and 0.9 ± 0.5 mm d^−1^); hot humid (HH, June–October, with Tmax, RH and PP of 31.4 ± 0.4 °C, 84.7 ± 1.0% and 7.8 ± 0.8 mm d^−1^); and fresh dry (FD, November–February, with Tmax, RH and PP of 26.8 ± 0.5 °C, 84.6 ± 0.3% and 0.7 ± 0.2 mm d^−1^) [18] in Veracruz, Mexico. At 19° 11′ N and 96° 20′ W, at 23 m amsl, with average annual temperature and precipitation of 24.8 °C and 1525.8 mm [15]. The climate of the region is AW0(w)(i’)gw’’, and hot sub humid with rains in summer [14].

### 2.2. Management of Donors

The study was carried out in accordance with the Official Mexican Standard (NOM-062-ZOO-1999) of technical specifications for the production, care and use of laboratory animals [27], as well as the regulations for the use and care of research animals from the Colegio de Postgraduados [28]. In total, 26 Romosinuano (RM) and 29 Tropical Milking (TM) non-lactating donors, nulliparous and up to three parturitions were selected to participate in this experiment. Ultrasonography exams of the reproductive tract were carried out to exclude animals with reproductive anomalies. No donor was breastfeeding. The RM and TM cows had a body weight (BW) of 429.3 ± 7.2 and 368.7 ± 17.8 kg, mean age of 95.9 ± 5.5 and 54.2 ± 4.9 months, and body condition score (BCS) of 2.6 ± 0.1 and 2.9 ± 0.1 (from Scale 1, emaciated, to Scale 5, obese [29]), respectively. The BC in HD, HH and FD was 2.6 ± 0.3, 2.9 ± 0.5 and 2.7 ± 0.4. In each climatic season, one OPU-IVF session was performed, with 10 (HD), 9 (HH) and 7 (FD) RM and 10 (HD), 9 (HH) and 10 (FD) TM cows. The donors were rotationally graze-fed only with para (*Brachiaria mutica* Forssk) and native grass (*Paspalum* spp.), were dewormed with Ivermectin (Virbamec^®^ Platinum, Virbac, Mexico City, Mexico) and received an application of phosphorus (Phospho ^®^ 20, Virbac, Mexico) monthly for four occasions, in addition to free mineral salt (Vimifos^®^, Mexico City, Mexico).

### 2.3. Follicular Aspiration and In Vitro Embryo Production

All reagents used for oocyte/embryo holding, manipulation and culture were purchased from Sigma^®^ Chemical Co (St. Louis, MO, USA) unless otherwise stated. For OPU, an ultrasound was used with a 7.5 MHz micro-convex transducer (Mindray^®^, DP-50Vet, Shenzhen, China) placed in an aspiration guide (WTA, Cravinhos, SP, Brazil). To aspirate the follicles, a 1 mm diameter × 120 cm plastic aspiration line and 20 G × 50 mm OPU needles were used, with an aspiration pressure of 80 mm Hg. The follicular fluid was collected in a 150 mL conical tube with a collection medium (Dulbecco’s Phosphate buffer, DPBS), supplemented with 1% fetal bovine serum (FBS) and 125 IU mL^−1^ of heparin at 37 °C [30].

The collected cumulus–oocyte complexes (COCs) were washed with DPBS and observed in a stereoscope (EMZ-8TR, Meiji Techno., Saitama, Japan). COCs were classified into four classes according to the number of cumulus cell layers and the characteristics of the ooplasm [31]. Grade 1, more than three complete layers of cumulus cells and uniform granulation, the ooplasm fills the zona pellucida uniformly (ZP); grade 2, less than three layers thick, ooplasm fills the ZP; grade 3, shrunken and degenerated ooplasm, far from the ZP with partial filling, and grade 4, naked oocytes, only enclosed by the ZP, in addition to presenting damage and remains of ooplasm [31]. 

Once classified, the COCs were grouped into viable (VIAB), including grades 1 and 2; unviable (UNV) as grade 3; and bare being considered as grade 4. Only the VIAB COCs were selected for subsequent maturation. The COCs were transported to the laboratory, located 30 min away, in 1.5 mL cryotubes with in vitro maturation medium (IVM); TCM-199, sodium bicarbonate with Earle’s salts (Gibco^TM^, Fisher Scientific, Madrid, Spain) supplemented with 10% FBS, 50 µg mL^−1^ gentamicin (Gentomicyn Super, Tornel, Mexico City, Mexico), 0.2 mM sodium pyruvate, 1 µg mL^−1^ estradiol 17-β (Benzoato de Estradiol Zoetis, Mexico), 10 µg mL^−1^ FSH (Folltropin-V, Bioniche Animal Health Canada Inc., Bellevile, ON, Canada), and 10 µg mL^−1^ hCG (Chorulon, MSD Intervet, Mexico) [32], in a lab mix portable incubator (WTA, Cravinhos, Sao Paulo, Brazil). In the laboratory, 10 COCs were placed in 50 μL drops of IVM, covered with mineral oil and transferred to the fixed bench incubator (WTA, 12419, Cravinhos, Sao Paulo, Brazil) to complete the 22 h period of IVM to 38 °C, 5% CO_2_ and saturation humidity. Once maturation was completed, the COCs were transferred to 90 μL drops of in vitro fertilization medium (IVFM); supplemented with 20 μL mL^−1^ of heparin and 6 mg mL^−1^ of essentially fatty acid-free BSA and covered with mineral oil [32].

IVF was carried out in the three seasons of the year, with the use, according to breed, of two high-fertility straws from RM and LT sires by season. The semen was prepared using a discontinuous Percoll gradient [33] and the concentration of 10^−6^ sperm mL^−1^ were used to fertilize each of the drops. After the 18 h IVF period, the presumptive zygotes were partially denuded with hyaluronidase, in a vortex for 5 min. In vitro culture medium (IVCM); KSOM, MR-107-D, supplemented with 10% FBS, 0.25 μg mL-1 gentamicin and 0.5% non-essential amino acids was carried out during seven days after IVF [32] when number and quality of the blastocysts were evaluated. Only code 1 embryos were cryopreserved by vitrification.

### 2.4. COCs Response Variables

Total COCs (COCsT) were observed in the field, with a stereoscope (EMZ-8TR, Meiji Techno., Saitama, Japan) in DPBS medium, when performing follicular aspiration, including VIAB, UNV, denuded (DE) and total matured in vitro (TMIV) with the cumulus cells expanded after the maturation period and the maintenance of cytoplasmic integrity [34,35].

### 2.5. Embryo Response Variables

In the embryos, on day two, after IVF, those cleavage embryos (CLI) with at least two cells were observed. Code one blastocysts (BC1), with a symmetrical and spherical internal cell mass with individual blastomeres similar in size, color and density with at least 85% of the cellular material intact; code two blastocysts (BC2) with minor irregularities in the inner cell mass or in size, color and density of individual cells and with at least 50% of their cell mass intact; total blastocysts (BLAST) all embryos at the blastocyst stage, including codes one and two, and degenerate blastocysts (DEG) with unviable cell mass [36].

### 2.6. Hormonal Covariates and Glucose

Two hormones were measured in each donor, basal insulin and serum cortisol, as well as serum glucose concentration. A blood sample was collected by puncture of the coccygeal vein, one day before each OPU at 6:00 h, with 21G × 38 mm needles and Vacutainer tubes (Vacutainer^®^, Becton Dickinson, Franklin Lakes, NJ, USA). Blood samples were collected and centrifuged for 15 min at 750× *g* at 4 °C. Serum samples were frozen and kept at −20 °C until further analysis. Basal insulin and serum cortisol concentrations were determined by chemiluminescence immunoassay (CLIA) (Maglumi 800; Shenzhen New Industries Biomedical Engineering Co., Ltd. (Snibe), Shenzhen, China), using commercial kits MAGLUMI^®^ insulin (CLIA, Shenzhen, China) and MAGLUMI^®^ cortisol (CLIA, Shenzhen, China), with intra- and inter-assay coefficient of variation (CV) of 36.9 and 37.4 to insulin and 77.7 and 81.1 to cortisol, respectively. Serum glucose concentration was determined by automated spectrophotometry with a clinical chemistry analyzer (Architect c4000; Abbott Diagnostics, Abbott Park, IL, USA) using a commercial reagent 3L82-20 with intra- and inter-assay CV of 9.4 and 9.3, respectively.

### 2.7. Physiological Covariates

The heart rate (HR) was measured in each donor, one day before the OPU at 15:00 h (hottest of the day), with a double bell stethoscope (BLESMED ^®^, Mexico City, Mexico), for one minute; the respiratory rate (RR), by observing the thoracic abdominal expansion on the left flank, for one minute and the rectal temperature (RT) with a veterinary digital thermometer (FlashCheck^®^, Delta Trak Inc., International, Mexico City, Mexico).

### 2.8. Statistical Analysis

The number of cows included per breed was determined by the availability of experimental units, for α = 0.01 and β = 0.90, taking into account the response variable VIAB. Linear fixed effects statistical models were used. The models included the factors of EST, breed and their interaction; body weight (BW) and age onset covariates were also included; the hormonal and glucose ones and the physiological ones of the hottest time. A P(λ) distribution of the experimental error was considered. Data were processed using generalized linear models and the GLM and GENMOD procedures of SAS [37]. 

## 3. Results

### 3.1. COCs

The EST x breed interaction affected all COC response variables (*p* ≤ 0.001; Table 1). The highest number of COCsT was observed in the HD and FD seasons and RM breed, higher for more than 10 in the lowest FD season and LT breed, with similar numbers in the other season and 9% more for RM. The highest number of VIABs occurred in FD and RM with more than 18 being the lowest in FD and TM; VIABs in both breeds were between 12 and 16 (*p* ≤ 0.001) and between seasons, from 12 to 15 (*p* > 0.05). The number of UNVs was close to zero in all seasons and breeds, except in the HH and TM, which was more than eight times higher than in HD and TM. The lowest number of DE occurred in the TM, close to four, while in the HD, with four times less than in FD, which had more DE than the HD and HH. The highest number of TMIV with more than 27 occurred in HD and RM and with less than 14 for TM in the same season; values close to 20 occurred in all other EST x breed combinations. RM had 8.5 more TMIV than TM.

A main effect of EST was observed on UNV (*p* ≤ 0.05) and DE (*p* ≤ 0.01), and of breed on VIAB (*p* ≤ 0.05) and TMIV (*p* ≤ 0.01). The lowest number of UNV was observed in HD, almost three times less than the highest number observed in HH. The number of DE was similar to six in HD and HH, but in FD, more than eleven were observed. Regarding breed, the VIAB and TMIV were higher in RM, with four and eight more compared to TM.

### 3.2. Effect of Hormonal and Physiological Covariates on COCs

Covariates affected COCs. BW affected all variables (*p* ≤ 0.01) except UNV, the betas were positive and similar, $\hat{\beta }$ = 0.002 ± 0.001 for COCsT, VIAB and DE and $\hat{\beta }$ = 0.004 ± 0.001 for TMIV. Age only had an effect on VIAB with $\hat{\beta }$ = 0.005 ± 0.002. Regarding the hormonal and glucose covariates, cortisol affected COCsT, VIAB and TMIV (*p* ≤ 0.01), the betas were all negative with the lowest $\hat{\beta }$ = −0.098 ± 0.014 for TMIV and the highest $\hat{\beta }$ = −0.045 ± 0.013 for COCsT. Insulin only affected COCsT (*p* ≤ 0.01), UNV (*p* ≤ 0.05) and DE (*p* ≤ 0.01), the betas were all negative, the lowest $\hat{\beta }$ = −0.251 ± 0.109 was observed in UNV and the highest $\hat{\beta }$ = −0.159 ± 0.032 in COCsT. Glucose significantly affected all the response variables of the COCs (*p* ≤ 0.01), except UNV. Glucose betas were all positive.

Physiological covariates affected COCs. HR affected COCsT, DE and TMIV (*p* ≤ 0.01), the lowest $\hat{\beta }$ = −0.020 ± 0.004, for TMIV and the highest $\hat{\beta }$ = −0.008 ± 0.003 for DE. Effect of RR was observed in COCsT, UNV and VIAB (*p* ≤ 0.01), the lowest $\hat{\beta }$ = −0.070 ± 0.021 for UNV and the highest $\hat{\beta }$ = -0.017 ± 0.004 for COCsT. RT only affected DE and VIAB (*p* ≤ 0.01), with beta negative for DE and positive for VIAB.

### 3.3. Embryos

The response in the embryonic variables due to the study factors is presented in Table 2. The EST x breed interaction only affected CLI (*p* ≤ 0.05), the greatest amount of CLI was observed in HD and RM, with more of nine than in the minor FD and TM; RM had twice as many CLIs as TM in all seasons. The main effect of EST was observed in BLAST (*p* ≤ 0.01) and DEG (*p* ≤ 0.05), the BLAST means were similar in HD and HH with values above three and lower in FD, with less than half compared to HD and HH. Regarding DEG, the lowest mean was observed in FD, similar to HH and nine times lower than HD, the highest. Breed only affected CLI (*p* ≤ 0.01); more than eleven were observed in RM, double the five observed in TM.

### 3.4. Effect of Hormonal and Physiological Covariates on Embryos

The covariates affected the embryos. BW affected DEG and BLAST (*p* ≤ 0.01), with $\hat{\beta }$ = −0.013 ± 0.004 and $\hat{\beta }$ = −0.004 ± 0.002, and age BC1 and BLAST (*p* ≤ 0.01), with $\hat{\beta }$ = 0.025 ± 0.006 and $\hat{\beta }$ = 0.015 ± 0.004. The hormones affected the embryos. Cortisol affected CLI (*p* ≤ 0.01), BC1 (*p* ≤ 0.01) and BLAST (*p* ≤ 0.05), negatively with the lowest $\hat{\beta }$ = −0.124 ± 0.053 for BC1 and the highest $\hat{\beta }$ = −0.081 ± 0.035 for BLAST. Insulin affected BC1 and BC2 (*p* ≤ 0.01), with $\hat{\beta }$ = −0.381 ± 0.132 for BC1 and $\hat{\beta }$ = −0.543 ± 0.232 for BC2. Glucose affected all embryo variables except BC2, with the lowest $\hat{\beta }$ = 0.201 ± 0.037 for BC1 and the highest $\hat{\beta }$ = 0.057 ± 0.014 for CLI. Physiological covariates also affected embryos. The HR affected CLI, BLAST and DEG (*p* ≤ 0.05), the lowest and positive $\hat{\beta }$ = 0.029 ± 0.014 was observed in DEG and the highest and negative $\hat{\beta }$ = −0.010 ± 0.005 in CLI. The RR affected CLI (*p* ≤ 0.05), BC1, BLAST and DEG (*p* ≤ 0.01); the betas were all negative except for CLI, with the lowest $\hat{\beta }$ = 0.154 ± 0.038 for DEG and the highest $\hat{\beta }$ = 0.015 ±0.007 for CLI. RT only affected BLAST (*p* ≤ 0.05) with $\hat{\beta }$ = −0.885 ± 0.427.

## 4. Discussion

It has been observed that, as the rectal temperature of cows subjected to heat stress increases, the uterine temperature increases, which affects the number of small and medium-sized follicles [38]. In this study, the lowest amount of COCsT, less than 20, was observed in the TM breed in the HD season, when the least precipitation occurs and the highest maximum temperatures are observed; however, the RM breed observed higher and similar numbers of COCsT. At the FD season, medium numbers of COCsT were observed with no difference between breeds (*p* ≤ 0.05; Table 1). In Brahman and Simmental × Brahman crossbreed heifers, a greater amount of COCsT and medium follicles (4.0 ± 0.2) useful in IVF were obtained in the fresh humid season and a lower amount in the hot dry (2.9 ± 0.2) [39].

In Black Japanese cows in the hot (28.3 ± 0.3 °C, 66.5% RH) and cool (17.5 °C, 62.9% RH) seasons, 11.2 ± 0.8 and 23.2 ± 1.9 COCsT were obtained by OPU [40] and in Gyr cows for 14 days in controlled environments at 25 and 38 °C, 11.2 ± 2.8 and 14.3 ± 2.5 COCsT were obtained [30]. In addition to climatological and environmental factors, genotype, aspiration frequency and semen used, among others, affect COCsT in an OPU-IVF program, [41]. In this study, for each kg of increase in BW of the donors, the COCsT increased with $\hat{\beta }$ = 0.002 ± 0.001; well-fed donors have greater energy reserves that are used in reproductive events [42]. On the other hand, as cortisol and insulin increased, COCsT decreased with $\hat{\beta }$ = −0.045 ± 0.013 and $\hat{\beta }$ = −0.159 ± 0.032. Heat stress in females activates the hypothalamic–pituitary–adrenal axis, which excites the pituitary gland to release the adrenocorticotropic hormone, which stimulates the release of glucocorticoids, such as cortisol, which when increased causes an inhibitory effect on the reproductive axis, which causes a delay in follicular development [43]. Furthermore, high concentrations of cortisol cause a decrease in glucose entry into cells, which result in high amounts of glucose and insulin in the blood [44]. Similarly, as the HR and RR increased, the COCsT decreased; increases in physiological covariates are presented as a mechanism generated by animals to release heat [45] and their higher frequencies indicate greater stress in the animal, which negatively affects its reproductive performance.

It is important to incubate only viable COCs, since correct IVM and subsequent IVF depend on it, as well as their development to the blastocyst stage [46,47]. The highest VIAB were observed in FD for RM and HH for TM (Table 1). The lowest temperatures in the region occur in FD, and although in HH, the Tmax and RH are high, the greatest precipitation also occurs, which allows the females to reduce body heat, with positive impact on the recovered VIAB. Increasing Tmax in HD directly affects follicular development and oocyte competence [48]. Our results differ from those by Torres-Júnior et al. [30] in Gyr cows, who observed no effect on VIAB (27.7 and 31.5%) from 25 °C to 38 °C environments. In this study, BW and age positively affected the COCsT, which may be related to the amount of VIAB; the highest COCsT occurred in FD and HH, and the highest precipitation also occurs in HH, so the availability of forage is greater in this season; donors with excellent body condition and BW correctly generate hormonal processes, allowing the development of follicles and oocytes of excellent quality [49]. On the other hand, although in other breeds the donor’s age affects the VIAB, the donors in this study had an age of 95.9 ± 5.5 in RM and 54.2 ± 4.9 months in LT; criollo breeds are characterized by their longevity, with cows that can remain productive for more than 15 years [23].

The greatest number of UNV was observed in HD and FD in TM; however, in these seasons, the greatest amount of COCsT was recovered, which may be related to the increase in UNV observed in TM (Table 1). In cows under heat stress, a lower quality of the COCs for IVF is observed. It has been found that oocytes recovered in hot seasons have a lower capacity to reach the blastocyst stage [50]. In cows under heat stress, the components of the cytoskeleton and mitochondrial functions and the correct expansion in cumulus cells are altered during maturation, which induces cell apoptosis [51] and requires two to three estrous cycles to recover good quality COCs damaged in the HD season [52]. In vitro matured studies have been carried out at 41 °C, to evaluate the damage caused by thermal shock in COCs and it has been observed that the damage in the ZP, with a greater number of pores, as well as abnormalities, increase after 22 h of exposure to high temperatures [53]. The cows in this study were kept exposed to grazing weather conditions throughout the year and the highest Tmax occurred in HD [18]. It has been observed that as heat stress increases, animals activate physiological mechanisms to release body heat [54]. In this study, as RR increased, UNV decreased with $\hat{\beta }$ = −0.070 ± 0.021, as product of the heat release that occurred in the donors as respiration increased.

Factors that are related to the increase in DE oocytes include the frequency and speed in the OPU and the gauge of the needle [55,56]. However, there are no previous studies showing the effect of season on the number of DE oocytes. In this study, a greater amount of DE was observed in HH and FD in TM and, although without statistical difference, greater amounts of DE in HD and HH in RM (Table 1). In Gyr cows, 15.8 and 23.0% DE oocytes were observed at low and high temperatures.

Hormonal covariates, insulin and glucose affected DE (Table 1); at greater increases in glucose, a greater amount of DE was observed. It is important to put at IVM those COCs with the greatest number of cumulus cells, since they allow their communication with the oocyte, influencing protein synthesis and gene expression for the subsequent expansion and maturation of the oocyte [35]. 

The highest TMIV means were observed in HD and HH for RM, but also the lowest in the same seasons for TM (Table 1). In zebu and crossbred cows, oocytes had a nuclear maturation of 76.9, 52.2 and 39.1% in the HH, HD and FD seasons [57]. In COCs subjected to heat shock of 41 °C in the first 12 h of maturation in vitro, oxidative stress and apoptosis were increased, the distribution of cortical granules was altered, the formation and potential of the mitochondrial membrane was reduced and damage was presented. In the cytoskeleton, reducing the cortical organization of actin [32] and the expansion of cumulus cells was reduced by 20% [51]. Andrew-Vázquez et al. [58] observed 14.5 times more abnormalities in the morphology of metaphase II oocytes subjected to heat shock of 41.5 °C in the first 3 h of in vitro maturation. As BW increased in the donors in this study, TMIV increased, which is related to the increase in VIAB as the females gained weight. It is advisable to maintain excellent nutrition in donors when undergoing reproductive biotechnologies; good nutrition generates better results [59]. In the donors of this study, as cortisol increased, TMIV decreased, high levels of cortisol occur as a response to heat stress [54], which in addition, caused an increase in HR and as a result, a lower amount of COCsT and VIAB. Therefore, it is advisable to expose only VIAB to in vitro maturation, as they have the highest probability of completing the process [47].

Among the factors that affect the division of the presumed zygotes are the quality of the semen used [60], the culture medium [61] and the quality of the oocyte collected, which can come from donors under heat stress [62]. In this study, the highest means of CLI embryos were observed in the HH for RM and FD for TM and the lowest in HD also for TM with almost 70% less in relation to the highest (Table 2). The highest temperatures occur in HD; it has been observed that more than six hours of exposure to extreme temperatures generates heat stress in females that decreases the cleavage of embryos, with a smaller number of embryos reaching at least eight cells [63] and a low percentage of embryos in the blastocyst stage [32].

Jersey embryos matured in vitro at 38.5 and 41 °C observed 11.4% less CLI at the highest temperature [64]. In Gyr cows, 74.6 and 80% of CLI were obtained at low and high temperatures [30]. Cortisol and glucose affected CLI in this study. As cortisol increased, CLI decreased. Large concentrations of cortisol indicate stress in the donors, which is reflected in lower quality oocytes that, although they complete the IVM, do not have the ability to cleavage. On the other hand, HR and RR affected CLI, the increases in heart and respiratory rates are used by animals as a heat release mechanism [45], so by increasing the flow of oxygenated blood, heat decreases, that in turn benefits the quality of the COCs and that affects the increase in CLI, as occurred in this study.

Efforts in in vitro embryo production focus on increasing BC1, since they have the greatest capacity to generate a pregnancy after being transferred [65] or survive cryopreservation [66]. In this study, the highest mean BC1 was observed in HH for both breeds, although without statistical differences (Table 2). The greatest precipitation occurs in the HH season, with greater availability and quality of forage [67]. However, the means of 1.4 ± 0.3 and 1.1 ± 0.3 for RM and TM were not different between breeds adapted to warm environments [26]. These *Bos taurus* breeds in adverse environments over the centuries developed adaptation characteristics such as slick hair coat [68] and mechanisms of metabolic heat reduction and increases in its dissipation through sweat glands and respiratory rate [69]. The age of the donor influenced the BC1, the females had a maximum of three births, so as the age increased, better development has been observed in the OPU that allows recovering COCs of better quality, without damage to the ZP and the greater number of cumulus cells, which allow the increase in BC1. The observed effect of the hormonal covariates and glucose is related to the increase in the heat stress of the donors [70]. As the hormones increased, the recovered COCs presented greater damage that was reflected in a decrease in BC1.

No effect of season or breed was observed on BC2 (Table 2); however, the means were very low, less than 0.8 ± 0.2. It is preferable to obtain BC1 and a smaller amount of BC2, since pregnancies are influenced by the quality of the blastocyst [65]. The increases in insulin in the donors decreased BC2; the high concentrations in the blood are related to the stress of the donors, since by reducing the entry of glucose into the cells, insulin concentrations increase as a mechanism protection and adaptation [71].

The quality of the oocyte is an important indicator of its development [46]; however, in hot climate tropical regions, exposure of females to more than 12 h of heat stress reduces the ability of embryos to reach the blastocyst stage [63]. The highest mean BLAST was observed in HD (3.6 ± 0.6; 45.5%), greater than HH (3.2 ± 0.5; 40.4%) and FD (1.4 ± 0.3; 24.9%). Criollo breeds are adapted to seasonal changes [23], since stressful conditions over decades generate genetic and metabolic adaptations that contribute to their physiological performance [72]. In Gyr cows [30], averages of 3.0 ± 0.5 and 1.7 ± 0.5 embryos were observed per donor at low and high temperatures. In RM cows [26], 30% and 18% BLAST were obtained in oocytes subjected to heat shock of 41 and 38.5 °C during IVM. On the other hand, Al-Katanani et al. [48] observed in BLAST derived from ovaries of Holstein cows, collected in winter and summer, 34.3 and 11.4% respectively, with a decrease of 22.9%. The global BLAST means obtained by season (Table 2) are similar to the three and four BLAST obtained by OPU-IVF in heifers and criollo cows from Ecuador [73,74].

Hormonal covariates, with the exception of insulin and physiological covariates, affected BLAST; these effects were also observed in VIAB. It is known that their quality has an impact on the development of embryos [47]. Small populations need the adoption of new technologies that facilitate the increase in pure and genetically superior animals [75]. In Colombia, a germplasm bank of criollo cattle has been created with embryos obtained in vivo and in vitro [76]; this activity can be of great support for the establishment of new pure cattle herds and the exchange between countries of criollo bovine germplasm of high genetic quality [77].

The greatest amount of DEG was observed in HD (1.1 ± 0.3), compared to HH (0.4 ± 0.1) and FD (0.2 ± 0.1), with no difference between breeds (Table 2). During the Veracruz HD season, there is less precipitation and greater solar radiation, with a Tmax of 31.6 ± 1.0 °C. High temperatures have been observed to induce DNA fragmentation and affect RNA levels [20,78]. Therefore, if COCs are exposed to high temperatures in hot seasons, the number of cells is reduced in the few embryos that reach the blastocyst stage, in addition to greater damage to the blastomeres [32] than increases the number of DEG embryos. The increase in BW of the donors in this study decreased the DEG; well-fed females allocate energy to reproductive events with better quality COCs. On the other hand, as glucose and HR increased, DEG increased, which is an indication of stress in donors [71].

## 5. Conclusions

Although body condition for healthy criollo cows under actual environmental conditions was similar in all three seasons, their exposure of criollo cows to the seasonal climatic variations of Veracruz and the physiological and hormonal changes had adverse effects on the cumulus–oocyte complexes and embryos. Although no effect of season and breed was observed in code one blastocysts, the number of degenerated embryos was greater in the hot dry season, so it can be concluded that the hot humid and cool dry seasons are the most favorable to perform OPU-IVF in Romosinuano and Tropical Milking cows.

## Figures and Tables

**Table 1:** Cumulus–oocyte complexes (COCs) collected by ovum pick-up in Romosinuano (RM) and Tropical Milking (TM) cows in three seasons in Veracruz, Mexico.

Variable	Season			
Breed	Hot Dry	Hot Humid	Fresh Dry	Global
Total COCs				
RM	29.6 ± 2.6 ^a^	22.6 ± 1.7 ^b^	29.1 ± 2.3 ^a^	26.9 ± 1.3
TM	18.9 ± 2.0 ^b^	28.4 ± 2.2 ^a^	26.6 ± 2.1 ^a^	24.2 ± 1.3
Global	23.6 ± 1.5	25.4 ± 1.4	27.8 ± 1.6	
Viable				
RM	17.3 ± 2.1 ^a^	12.8 ± 1.3 ^b^	18.7 ± 1.9 ^a^	16.1 ± 1.0 ^A^
TM	13.7 ± 1.8 ^ab^	17.4 ± 1.8 ^a^	8.0 ± 1.0 ^c^	12.4 ± 0.9 ^B^
Global	15.4 ± 1.2	14.9 ± 1.1	12.2 ± 1.0	
Unviable				
RM	1.7 ± 0.6 ^b^	1.7 ± 0.5 ^b^	1.8 ± 0.5 ^b^	1.8 ± 0.3
TM	0.4 ± 0.3 ^a^	3.0 ± 0.8 ^b^	1.5 ± 0.5 ^b^	1.2 ± 0.3
Global	0.9 ± 0.3 ^Z^	2.3 ± 0.4 ^XY^	1.7 ± 0.4 ^YZ^	
Denuded				
RM	9.4 ± 1.4 ^b^	7.5 ± 1.0 ^b^	8.3 ± 1.1 ^b^	8.4 ± 0.7
TM	4.2 ± 0.8 ^a^	6.0 ± 1.0 ^ab^	17.1 ± 2.2 ^c^	9.6 ± 0.7
Global	6.3 ± 0.7 ^X^	6.7 ± 0.7 ^X^	11.9 ± 1.1 ^Y^	
Total matured in vitro				
RM	27.8 ± 2.7 ^a^	24.6 ± 2.0 ^a^	22.9 ± 2.1 ^a^	25.0 ± 1.3 ^A^
TM	13.3 ± 1.5 ^c^	16.7 ± 1.5 ^bc^	20.0 ± 1.8 ^ab^	16.5 ± 1.0 ^B^
Global	19.3 ± 1.3	20.3 ± 1.2	21.4 ± 1.4	

^A,B^ Different literal per row indicates statistical difference (*p* ≤ 0.01). ^X,Y,Z^ Different literal per column indicates statistical difference (*p* ≤ 0.006). ^a,b,c^ Different literal per column for season and row for breed indicates statistical difference (*p* ≤ 0.01).

**Table 2:** Embryos produced in vitro (IVF) from Romosinuano (RM) and Tropical Milking (TM) cows in three seasons in Veracruz, México.

Variable	Season			
Breed	Hot dry	Hot humid	Fresh dry	Global
Cleavage				
RM	10.8 ± 1.6 ^ab^	13.3 ± 1.7 ^a^	10.8 ± 1.5 ^ab^	11.6 ± 1.0 ^A^
TM	3.9 ± 0.7 ^c^	5.4 ± 0.9 ^bc^	7.8 ± 1.2 ^b^	5.5 ± 0.6 ^B^
Global	6.5 ± 0.7	8.5 ± 0.8	9.2 ± 0.9	
Blastocysts code one				
RM	1.5 ± 0.5	1.6 ± 0.5	1.1 ± 0.4	1.4 ± 0.3
TM	1.3 ± 0.6	1.5 ± 0.4	0.6 ± 0.2	1.1 ± 0.3
Global	1.4 ± 0.4	1.5 ± 0.3	0.9 ± 0.2	
Blastocysts code two				
RM	1.3 ± 0.7	0.7 ± 0.4	0.4 ± 0.2	0.7 ± 0.2
LT	0.5 ± 0.3	0.5 ± 0.3	0.3 ± 0.2	0.4 ± 0.2
Global	0.8 ± 0.3	0.6 ± 0.2	0.4 ± 0.2	
Total blastocysts				
RM	5.0 ± 1.2	3.4 ± 0.8	1.8 ± 0.5	3.1 ± 0.5
TM	2.6 ± 0.7	3.0 ± 0.6	1.1 ± 0.3	2.1 ± 0.4
Global	3.6 ± 0.6 ^X^	3.2 ± 0.5 ^X^	1.4 ± 0.3 ^Y^	
Degenerate				
RM	2.0 ± 0.9	0.3 ± 0.1	0.2 ± 0.1	0.5 ± 0.2
TM	0.6 ± 0.3	0.7 ± 0.3	0.2 ± 0.1	0.4 ± 0.2
Global	1.1 ± 0.3 ^X^	0.4 ± 0.1 ^XY^	0.2 ± 0.1 ^Y^	

^A,B^ Different literal per row indicates statistical difference (*p* ≤ 0.01). ^X.Y^ Different literal per column indicates statistical difference (*p* ≤ 0.03). ^a,b,c^ Different literal per column for season and row for breed indicates statistical difference (*p* ≤ 0.02).

## Data Availability

The raw data supporting the conclusions of this article will be made available by the authors on request.
